# Management and prognostic prediction of appendiceal mucinous adenocarcinoma with peritoneal metastasis: a single center study in China

**DOI:** 10.1186/s12885-020-06787-4

**Published:** 2020-04-06

**Authors:** Ruiqing Ma, Bing Wang, Xichao Zhai, Yiyan Lu, Hongbin Xu

**Affiliations:** 1 Department of Myxoma, Aerospace Center Hospital, Beijing, 100049 China; 2 Department of Pathology, Aerospace Center Hospital, Beijing, China

**Keywords:** Appendiceal mucinous adenocarcinoma, Pseudomyxoma peritonei, Completeness of cytoreduction score, Prognosis

## Abstract

**Background:**

To investigate the clinical and pathological characteristics of appendiceal mucinous adenocarcinoma with peritoneal metastasis and analyze the prognostic factors.

**Methods:**

A retrospective analyses of clinicopathological features of 50 patients with appendiceal mucinous adenocarcinoma with peritoneal metastasis from January, 2013 to December, 2017 in Aerospace Central Hospital, Beijing, China. Survival data calculation and comparison were respectively performed with the Kaplan-Meier method and the log-rank test. The Cox proportional hazards regression method was used for multivariate survival analyses.

**Results:**

Cytoreduction for appendiceal mucinous adenocarcinoma was conducted on 50 patients (24 males and 26 females), with a median age of 52.5 years at the time of surgery (range 31–71 years). The median overall survival (OS) time was 24 months, with 2-,3- and 5-year survival rates of 53, 24 and 8%, respectively. At the last follow-up in December 2018, 13 patients were still alive. Multivariate analysis revealed that patients who had low Ki-67 expression (less than 50%) and CCR (completeness of cytoreduction) 0/1/2 score had significantly better OS rate than their respective counterparts.

**Conclusions:**

Ki-67 expression statue and CCR score could be employed as the prognosis prediction in patients with appendiceal mucinous adenocarcinoma.

## Introduction

Malignant appendix neoplasms are extremely rare and the incidence of primary appendiceal cancers was less than 1.5% (approximately 0.12 per 1,000,000 person years) according to the appendectomy specimens examination [1–3]. As the primary malignant neoplasms of the appendix, appendix adenocarcinomas includes mucinous, non-mucinous (colonic-type), and signet-ring cell adenocarcinomas [4]. Among them, mucinous adenocarcinoma is the most common type. Since appendix carcinomas may cause appendicitis or the appendix rupture, and pseudomyxoma peritonei (PMP) could be presented as a typical clinical syndrome when mucinous adenocarcinoma of the appendix spreads to the peritoneal cavity, which is characterized by a large amount recurrent and recalcitrant mucinous ascites caused by surface growth on the peritoneum without obvious underlying tissues invasion.

According to the 8th edition of the American Joint Committee on Cancer (AJCC) Staging Manual, mucinous adenocarcinomas of the appendix are classified into low-grade and high-grade (well-differentiated and moderately/poorly differentiated, respectively) tumor based on histological grade in the AJCC TNM Staging System [5–7]. Due to the low incidence, lacking of available data about the prognostic factors for appendix mucinous adenocarcinomas and non-adenocarcinomas in current clinical practice.

Here, we presented our single center data about the clinical and pathological characteristics of appendiceal mucinous adenocarcinoma and analyze the prognostic factors by retrospective review of 50 cases of patients.

## Patients and methods

### Ethical approval of the study

The study was approved by the Ethic committee of Aerospace Central Hospital, Beijing, China (no.20161109-ST-07). Written inform consent was obtained from all the included patients, and all the participants have given the consent for their images.

### Patients

A retrospective analyses of a cohort of appendiceal mucinous adenocarcinoma patients with peritoneal metastasis from January, 2013 to December, 2017 in the Department of Myxoma of Aerospace Central Hospital, Beijing, China. All the patients were screened from high grade peritoneal pseudomyxoma and confirmed by 2 experienced pathologists. Mucinous adenocarcinoma was featured by invasive peritoneal lesions comprised with abundant glandular or signet ring cell morphology epithelium with sufficient architectural complexity and cytological atypia to a diagnosis confirmation of mucinous carcinoma. The diagnosis criteria were according to the 8th Edition of American Joint Committee on Cancer [7]. The exclusion criteria were as follows: 1. Lost follow-up; 2 combined with other severe organic disease; 3. with incomplete disease history. Finally, a total of 50 patients were included for analyses.

### Surgical procedure

**Fig. 1:**
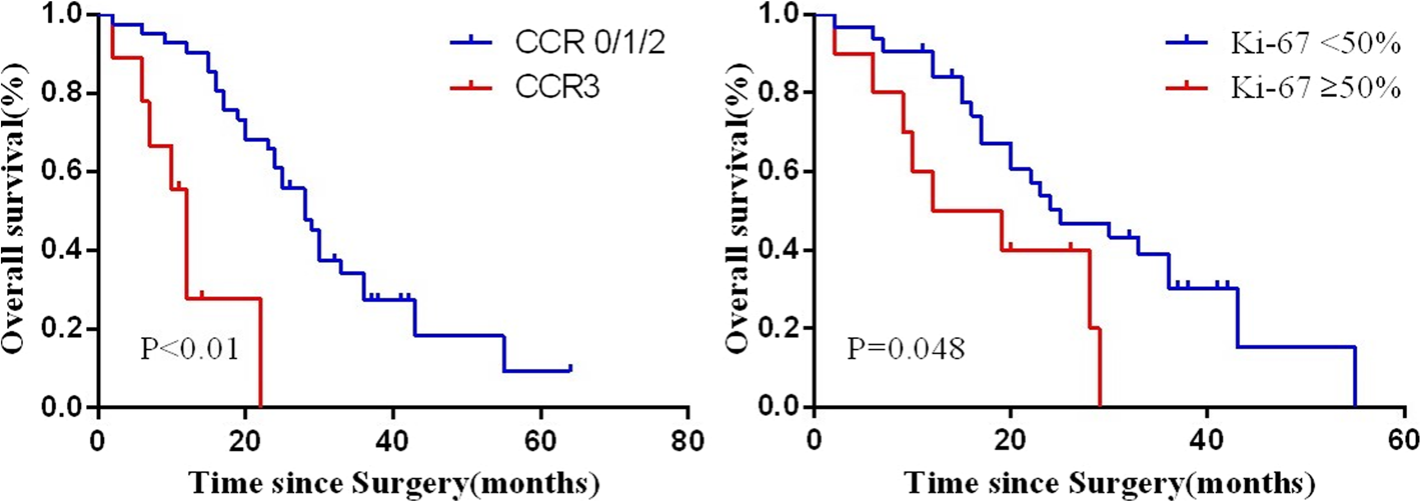
Surgical procedure and pathological analysis of appendiceal mucinous adenocarcinoma. **a**. Mucinous ascites in peritoneal cavity (black arrow); **b**. Great omentum cake in peritoneal cavity; **c**. The status of abdominal cavity after cytoreduction therapy; **d**. CT scan at the time of pre-cytoreduction therapy (black arrow indicated the lesion); **e**. CT scan image after cytoreduction therapy (black arrow indicated no lesion postsurgery); **f**. H&E staining of tissue sections of appendiceal mucinous adenocarcinoma

Sugarbaker technique of cytoreductive surgery (CRS)/ hyperthermic intraperitoneal chemotherapy (HIPEC) described previously were employed here [8]. Briefly, aggressive cytoreduction was employed to remove as much macroscopic disease as possible. HIPEC is administered at thecompletion of cytoreduction using closed technique. Two inflow and two outflow catheters were allocated in the abdomen. After temporary the abdominal skin closure, mitomycin at the dosage of 20 mg/m^2^ was performed to increase to an inflow temperature of 41 to 42 °C and follow by a 60 min’ peritoneal cavity circulation (Fig. 1).

### Data collection

The interested variables included age of the patient at the time of diagnosis, sex, Prior surgical score (PSS), with/without preoperative intravenous chemotherapy, preoperative intraperitoneal chemotherapy, hemoglobin levels, albumin levels at the time of admission, preoperative CEA/CA125/CA199 elevation, Intraoperative peritoneal cancer index (PCI), completeness of cytoreduction (CCR) score, intraoperative HIPEC; pathological data include: with/without lymph node metastasis, presence or absence of signet ring cells. Among these characteristics, PSS was scored 0–3, and PSS-0: biopsy only, PSS-1: minimal prior dissection with only one dissected abdominal region; PSS-2: 2–5 regions dissected and PSS-3: extensive prior cytoreduction (more than 5 regions). PCI stands for a clinical integration of both peritoneal implant size and distribution of peritoneal surface malignancy [9]. For PCI evaluation, 13 anatomical regions were divide based on the region of the abdomen and pelvis. The largest size malignant nodule at each region was scored and the lesion size score from the all 13 regions was summated as the PCI for the individual patient [8]. CCR score was recorded at the time of the completion of the surgical procedure. CCR-0: no peritoneal seeding was visible after the cytoreduction, CCR-1: residual tumour nodules less than 2.5 mm, CCR-2: residual tumour nodules 2.5 mm to 2.5 cm and a CCR-3: larger than 2.5 cm persistent tumor nodules [10].

### Statistical analyses

Statistical analyses were employed using SPSS 20.0(SPSS Inc. Chicago, IL). Continuous data were expressed as medians and range. Categorical variables were presented as number and percentages. The associations between the clinicopathological variables and survival data was identified by univariate and multivariate Cox proportional hazard regression models. All variables with *p* value < 0.3 in the univariate analysis were further analyzed with in the multivariate Cox model. Survival data calculation and comparison were respectively performed with the Kaplan-Meier method and the log-rank test. Statistically significance was considered when the presence of two tailed *P* values < 0.05.

## Results

### Demographic and pathological characteristics of patients

Cytoreductive surgery for appendiceal mucinous adenocarcinoma was carried out on 50 patients (26 males and 24 females), with a median age of 52.5 years at the time of surgery (range 31–71 years). The median survival rate was 24 months, whereas 2-,3- and 5-year survival rates of 53, 24 and 8%, respectively. Prior systemic chemotherapy was performed in 13(26%) patients, whereas 15(30%) patients were treated with HIPEC. Forty-eight (96%) patients were with bloating or ascites or abdominal mass manifestation. Comorbidities including ileus, anemia and hypoalbuminemia were respectively found in 18(36%), 23(46%) and 9(18%) patients. Increased levels of Serum tumor maker CEA, CA125 and CA199 were respectively in 33(66%), 34(68%) and 27(54%) patients. The number of patients with PCI score > 26, 13–26, and < 13 were respectively 34(68%), 14(28%) and 2(4%). Demographic and pathological characteristics of patients were listed in Table 1.

**Table 1:** Patient demographic data(*n* = 50)

Characteristics	
Gender	
Female	24(48%)
Male	26(52%)
Disease duration (median; range; months)	18(1–357)
PSC	
No	37(74%)
Yes	13(26%)
PPC	
No	35(70%)
Yes	15(30%)
Manifestation at diagnosis	
Bloating or ascites or abdominal mass	48(96%)
Others	2(4%)
Comorbidities	
ileus	18(36%)
anemia	23(46%)
hypoalbuminemia	9(18%)
Increased levels of Serum tumor maker	
CEA	33(66%)
CA125	34(68%)
CA199	27(54%)
PCI	
<13	2(4%)
13–26	14(28%)
>26	34(68%)
CCR	
0,1	20(40%)
2	21(42%)
3	9(18%)
HIPEC	
No	9(18%)
Yes	41(82%)
Signet ring cell	
No	42(84%)
Yes	8(16%)
Lymph node metastasis	
No	43(86%)
Yes	7(14%)
Ki-76	
<50	32(64%)
≥ 50	18(36%)
Follow-up time (median; range; months)	24(12–36)

*PSC* Previous systemic chemotherapy, *PPC* previous peritoneal chemotherapy, *PCI* peritoneal cancer index, *CCR* completeness of cytoreduction, *HIPEC* Hyperthermic intraperitoneal chemotherapy

### Multivariable cox regression analysis for prognosis prediction

According to the multivariate analysis, significantly better overall survival rate was found in the patients with a low Ki-67 expression (less than 50%) and CCR0/1/2 score than their corresponding counterparts (Table 2).

**Table 2:** Univariate and multivariable Cox Proportional Hazards Regression Model for prognosis prediction in patients with appendiceal mucinous adenocarcinoma

Variable	Univariate hazard ratio(range)	*P* value	Multivariate hazard ratio(range)	*P* value
Age (> 60 vs. ≤ 60)	1.059 (0.545–2.056)	0.867		
Gender	1.334 (0.683–2.603)	0.399		
PSS	0.898 (0.663–1.216)	0.487		
CA125	1.873 (0.877–4.000)	0.105		
PSC	0.573 (0.249–1.319)	0.191		
Ileus	0.703 (0.342–1.445)	0.338		
PCI	1.836 (0.942–3.576)	0.074		
CCR (0,1,2 vs.3)	6.962 (2.578–18.800)	< 0.001	9.785 (3.381–28.318)	< 0.001
HIPEC	0.758 (0.314–1.832)	0.538		
Ki-67 expression (< 50% vs. ≥ 50%)	1.715 (0.856–3.438)	0.128	2.356 (1.125–4.931)	0.023
Signet ring Cell	2.073 (0.895–4.802)	0.089		
Lymph node metastasis	1.874 (0.638–5.507)	0.253		

*PSS* prior surgical score, *PSC* Previous systemic chemotherapy, *PCI* peritoneal cancer index, *CCR* completeness of cytoreduction, *HIPEC* Hyperthermic intraperitoneal chemotherapy

### Survival

**Fig. 2:**
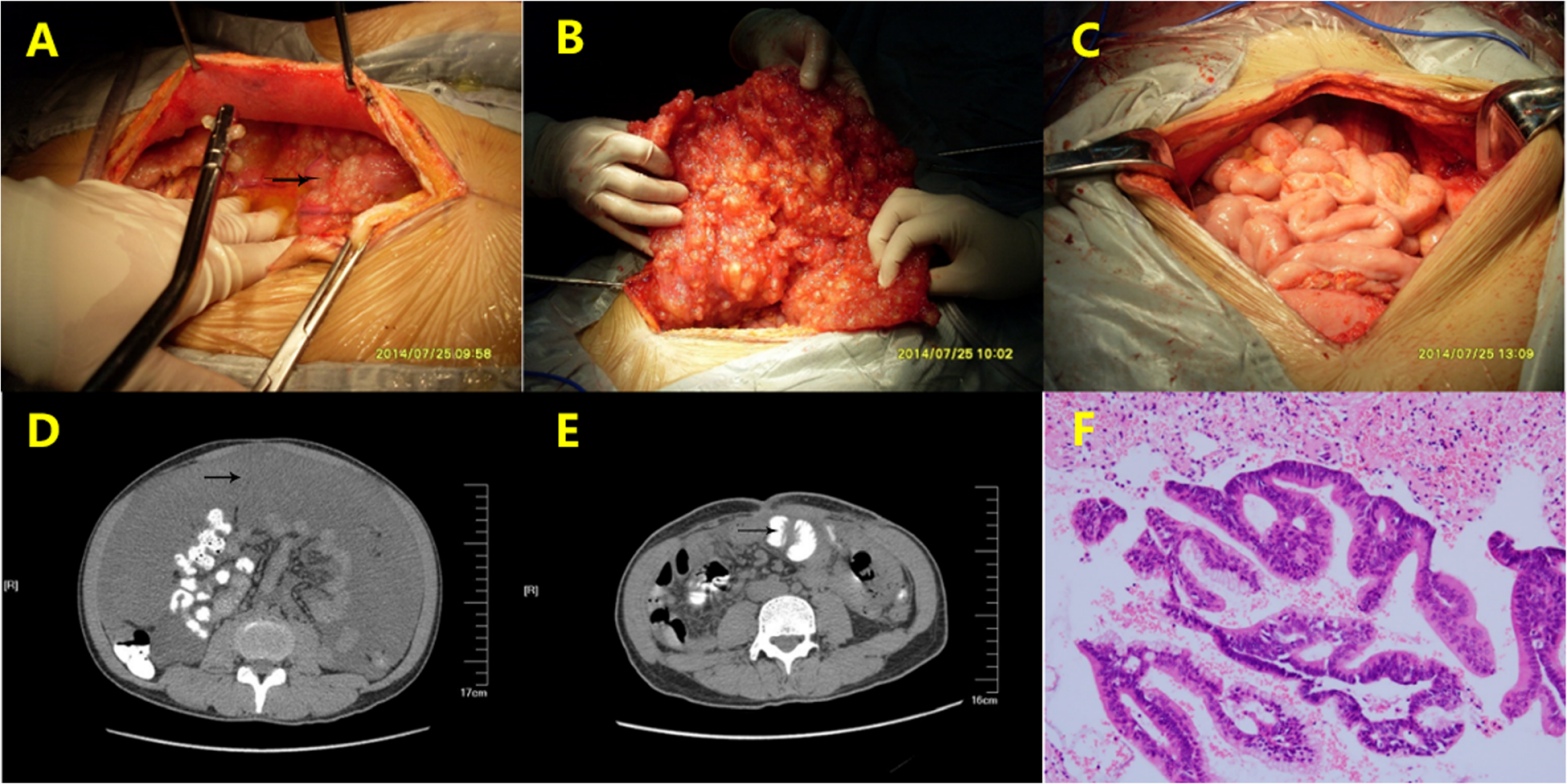
Kaplan-Meier survival curve according to the completeness of cytoreduction (CCR) classification and Ki-67 classification

At the last follow-up in December 2018, 13 patients were still alive. The median overall survival (OS) time was 24 months, while the 2-, 3- and 5-year survival rates of 53, 24 and 8%, respectively. The 1-year, 2-year survival rates for CCR0/1/2 and CCR3 were 90% vs 28, 66% vs 0%, respectively. The 1-year, 2-year survival rates for Ki-67<50% and Ki-67 ≥ 50% were 67% vs 50, 39% vs 0%, respectively (Fig. 2).

## Discussion

In present study, we performed a retrospective analysis of clinicopathological features of 50 patients with appendiceal mucinous adenocarcinoma to investigate the possible prognosis related factors. Our results indicated that cytoreduction for appendiceal mucinous adenocarcinoma was conducted on 50 patients (24 males and 26 females), with a median age of 52.5 years at the time of surgery (range 31–71 years).

Immediate progression was considered in the majority of patients who underwent debulking surgery (CCR2/3). Only 20 patients had CCR0/1 cytoreduction. So, progression-free survival was not discussed in this study. The median overall survival (OS) time was 24 months, while the 2-, 3- and 5-year survival rates of 53, 24 and 8%, respectively. At the last follow-up in December 2018, 13 patients were still alive. Multivariate analysis revealed that patients who had less than 50% Ki-67 expression and CCR0/1/2 score had significantly better OS rate than their respective counterparts.

Mucinous adenocarcinomas are considered as the unique appendix tumours based on their particular biological behavior. Among different types of appendiceal neoplasms, greater potential of the presence of serosa invasion and peritoneum or abdominal cavity spreading and followed by PMP formation was found in the mucinous adenocarcinomas. As a rare clinical disease, the characteristics of PMP include excessive accumulation of gel-like mucinous peritoneal fluid in the peritoneal or pelvic cavity, thereby resulting in clinical manifestation such as abdominal pain, abdominal mass, progressive increasing of the abdominal circumference and severe weight loss [11, 12]. Variable results were found on the survival rate of patients with mucinous appendiceal adenocarcinoma according to previous reports [13–15]. Overman et al*.* found that stage IV diseases were more likely to be presented as mucinous adenocarcinomas than those with non-mucinous adenocarcinomas [16]. Therefore, the subdivision of the patients into Stage I-III and IV was conducted by Xie et al., and their results showed that significantly decreased 5-year overall survival was found in patients with mucinous adenocarcinoma, which indicated the different biological behaviors between mucinous and non-mucinous adenocarcinomas.

Multidisciplinary therapies employing surgical resection followed by adjuvant chemoradiation treatment, have been increasingly performed as the treatment modality in patients with resectable digestive tract cancers [17, 18]. However, controversy was remained existed about whether the multidisciplinary therapies could result in a survival improve effects in patients with adenocarcinoma of the appendix. Since the development of PMP was the frequently presented complications in patients with mucinous adenocarcinomas of the appendix could, cytoreductive surgery and HIPEC have been suggested as first-line therapies [19]. Recently, Asare et al demonstrated that systematic chemotherapy could lead to a significantly improved OS without considering the history feature of the adenocarcinoma (HR:0.79; 95% CI:0.69–0.90; *P =* 0.0005 and HR:0.84; 95% CI: 0.75–0.95; *P =* 0.004 for mucinous and nonmucinous, respectively). Moreover, systemic chemotherapy seems to have no effects on the patients with mucinous and well-differentiated adenocarcinomas [20, 21]. In our study, multivariate analysis revealed that patients with CCR0/1/2 score had significantly improved 5-year overall survival rate than those with CCR3, whereas The OS times for the cohort based on CCR score of tumors were 28 months and 12 months, respectively, for CCR0/1/2, and CCR3. These results also supported the effectiveness of HIPEC in the treatment of mucinous appendicular adenocarcinoma.

There are also some limitations in our study. Firstly, this is a single center small size study and insufficient sample size could affect the final conclusion. Secondly, the diagnosis value of Ki-67 expression was also implicated by the results obtained here, and although we calculated accurate cut-off value of Ki-67 level by ROC curve, lacking of comparison results with golden standard could result in failure to elucidate the diagnostic efficacy of Ki-67. Third, the detailed mechanisms that resulted in the efficacy of CRS should be elucidated for identification of the exact role of CRS in mucinous appendicular adenocarcinoma. Taken together, further investigation is still important to identify the biomarker role of CCR scores by a multicenter clinical large sample size study with prognosis results.

## Conclusions

In conclusions, we demonstrated that Ki-67 expression statue and CCR score could be employed as the prognosis prediction in patients with appendiceal mucinous adenocarcinoma. Any effort to achieve CCR0/1/2 by operation is valuable to improve the prognosis. Patients with high volume disease such as appendiceal mucinous adenocarcinoma should be examined in a specialized center for evaluation based on the consideration of the potential survival benefit that may be achieved after cytoreduction.

## Data Availability

The datasets used and/or analyzed during the current study are available from the corresponding author on reasonable request.

## Abbreviations

OS: overall survival
CCR: completeness of cytoreduction
PMP: pseudomyxoma peritonei
AJCC: American Joint Committee on Cancer
CRS: cytoreductive surgery
HIPEC: hyperthermic intraperitoneal chemotherapy
PSS: prior surgical score
PCI: peritoneal cancer index
